# The values attributed by physicians and nurses to abortion in Brazil

**DOI:** 10.1590/0034-7167-2024-0196

**Published:** 2025-03-31

**Authors:** Ana Zélia Silva Fernandes de Sousa, Valdecyr Herdy Alves, Andressa Tavares Parente, Bianca Dargam Gomes Vieira, Audrey Vidal Pereira, Lucia Helena Garcia Penna, Giovanna Rosario Soanno Marchiori, Diego Pereira Rodrigues

**Affiliations:** I Universidade do Estado do Pará. Tucuruí, Pará, Brazil; II Universidade Federal Fluminense. Niterói, Rio de Janeiro, Brazil; III Universidade Federal do Pará. Belém, Pará, Brazil; IV Universidade do Estado do Rio de Janeiro. Rio de Janeiro, Rio de Janeiro, Brazil; V Universidade Federal de Roraima. Roraima, Boa Vista, Brazil

**Keywords:** Women, Obstetrics, Abortion, Legal, Health Personnel, Reproductive Rights., Mujeres, Obstetricia, Aborto Legal, Personal de Salud, Derechos Sexuales y Reproductivos.

## Abstract

**Objectives:**

to understand the values attributed by nurses and physicians working in obstetric care for abortion.

**Methods:**

this is a phenomenological study based on Max Scheler’s theory of values, with 19 semi-structured online interviews with healthcare professionals working in legal abortion, using the snowball sampling recruitment technique. The collected data were transcribed in full and submitted to content analysis.

**Results:**

it was evident from healthcare professionals’ statements that abortion has a vital, moral and ethical professional value. Furthermore, the lack of value given to abortion by physicians and nurses was demonstrated as well as the vital value attributed to women’s lives by public health.

**Final Considerations:**

in order to understand the values attributed by nurses and physicians, there was the need to advance the values of health, rights, dignity and respect for women in the face of abortion.

## RESUMEN

**Objetivos:**

comprender los valores atribuidos por enfermeras y médicos que actúan en la atención del aborto obstétrico.

**Métodos:**

se trata de un estudio fenomenológico basado en la teoría de valores de Max Scheler, con 19 entrevistas semiestructuradas en línea a profesionales de la salud que trabajan en el aborto legal, utilizando la técnica de reclutamiento por muestreo de bola de nieve. Los datos recopilados fueron transcritos en su totalidad y sometidos a análisis de contenido.

**Resultados:**

se evidenció, a partir de las declaraciones de los profesionales de la salud, que el aborto tiene un valor ético vital, moral y profesional. Además, quedó demostrada la infravaloración del aborto por parte de médicos y enfermeras, así como el valor vital que la salud pública atribuye a la vida de las mujeres.

**Consideraciones Finales:**

para comprender los valores atribuidos por enfermeras y médicos, se mostró la necesidad de avanzar en los valores de salud, derechos, dignidad y respeto a las mujeres frente al aborto.

## INTRODUCTION

Maternal deaths resulting from abortion in Brazil occupy a prominent position in the obstetric scenario. The consequences of abortions are mainly noted in women in more vulnerable socioeconomic conditions. Specialized literature indicates that, in developing countries, such as those in Latin America, Asia and Africa, there is a higher risk of death due to unsafe abortions compared to those performed in developed countries. Developed countries, such as the United States of America, France and Germany, have more modern methods as well as legislation that supports abortion from a non-punitive perspective. Thus, the advancement of legal and legal aspects for legal abortion becomes a value in the current discussion for Brazilian society^(1-8)^. According to a survey by the World Health Organization (WHO), only half of registered abortions in the world are performed using the recommended method^(4)^.

The WHO^(9)^ estimates that 22 million unsafe abortions occur worldwide each year, resulting in approximately 47,000 deaths among women. Based on data available on the Brazilian Healthcare system (Portuguese acronym: SUS) Department of Information Technology website, in the first half of 2020, the number of procedures performed in SUS due to complications after abortions exceeded by 79 times the number of gestational interruptions provided for by law^(2,10)^, demonstrating that it is a public health concern.

The study conducted a survey using the Live Birth Information System and the Mortality Information System between 2006 and 2015. A total of 770 maternal deaths with the underlying cause of abortion were found in the Mortality Information System. Unspecified abortion remained the most frequent underlying cause among deaths due to abortion during the period assessed, with an average of 56.5% of cases. Among the 770 deaths with the underlying cause declared as abortion, only 0.9% of deaths were due to abortion for medical and legal reasons; 14.9% were declared as spontaneous abortions; 15.2% were declared as other types of abortion; and 12.5% were declared as failed abortion attempts^(5)^.

In Brazil, abortion is a crime provided for in the Penal Code in Articles 124, 125 and 126, with penalties for women and physicians who perform it. However, since 1940, Brazilian jurisprudence has provided for legalized abortion for: 1) pregnancies resulting from rape (preceded by consent of pregnant women or, when incapacitated, of their legal representative); 2) in cases where there is no other way to save pregnant women’s lives, due to their risk of death; and 3) recently, in 2012, through the Action Against a Violation of a Constitutional Fundamental Right (Portuguese acronym: ADPF) 54, the Brazilian Supreme Federal Court (Portuguese acronym: STF) added the pregnancy of a fetus with anencephaly as a condition for termination of pregnancy, providing these legal alternatives for its authorization^(7,11)^.

In this regard, legal or safe abortion, which constitutes the termination of pregnancy permitted by law, must be performed only by qualified professionals, with the support of regulations, public policies and adequate infrastructure of healthcare systems, providing supplies, equipment and care flows so that women have access to these services. Failure to implement favorable conditions represents an attack on Brazilian women’s lives and health, requiring reproductive justice due to the government’s disregard for ensuring their right as a value^(1,3,12)^.

Abortion provided for by law is seen by healthcare professionals as a right that women have won, although some are (un)aware of legislation and remain away from this process due to their opposing moral, religious or ideological position. In healthcare services where legal abortion is available, professionals are noted to be insufficiently qualified on the subject and to be out of line with the Ministry of Health technical standards, evidencing such a gap in their performance^(13)^.

Thus, in 2022, the WHO^(4)^ presented updated guidelines on protecting women’s and girls’ health in relation to abortions, aiming to present recommendations and best practices regarding the procedure, aiming to enable quality evidence-based global care. Among the guidelines, the importance of strengthening information dissemination on contraceptive methods stands out, the urgency of removing existing unnecessary political barriers, such as criminalization, mandatory waiting periods, family consent or even other ways of preventing or delaying the procedure, decriminalizing abortion and making it accessible to girls and women.

Understanding the values of healthcare professionals working in obstetric care is essential to promote practices that ensure abortion is performed without disrespecting women. It is essential to seek reproductive justice in relation to abortion, expanding human rights and pursuing social equity^(12)^. The reality of work in performing legal abortions must imply a transformation in the care provided to the country, highlighting the importance of effective care for women, considering their feelings, affections and subjectivity arising from the situation. This includes the defense of sexual, reproductive and human rights, avoiding State negligence in relation to abortion.

In this context, it is necessary, according to Max Scheler’s perspective, to reflect on the concept of value. For the philosopher, everything that is relevant to a person’s life is considered value. Each value is constructed by the subject and aims to meet their needs, i.e., a value becomes significant when it contributes to meeting a person’s experiential and existential needs^(14)^. Other concepts present in the theory of values refer to countervalue and non-value; the first opposes value, whereas the second expresses indifference towards it.

Thus, healthcare professionals’ values regarding abortion may vary. Some attribute a positive value to life and ethics, whereas others express counter-values or indifference towards abortion^(14)^. When caring for women undergoing abortion, it is essential that professionals are attentive to women’s lives, considering them an essential value that must be guided by respect, dignity and social and reproductive justice.

In this regard, the study had as its guiding question: what values do nurses and physicians working in obstetric care attribute to abortion?

Thus, training healthcare professionals to assist women in abortion situations and providing access to abortions as provided for in Brazilian law, with the provision of specialized services, are essential to guarantee the SUS precepts and Brazilian women’s values and rights. Scientific literature shows the moral, ethical, religious, legal and cultural conflict in legal abortion, and, in the field of professional practice, it becomes relevant to bring to light the problem of inaction in the face of the realization of the rights guaranteed to women. Thus, these impacts are reflected in the impartiality of the care provided, loaded by healthcare professionals with judgment, prejudice and disbelief in the reports presented, weakening the quality and safety of the care provided to women seeking legal termination of pregnancy^(15-17)^.

## OBJECTIVES

To understand the values attributed by nurses and physicians working in obstetric care for abortion.

## METHODS

### Ethical aspects

The study was conducted in accordance with national and international ethics guidelines, and was approved by the *Universidade Federal do Pará* Institute of Health Sciences (IHS) Research Ethics Committee, according to Protocol 5.712.770/2022, as provided for in the Brazilian National Health Council (Portuguese acronym: CNS) Resolution 466/2012.

Voluntary participation was guaranteed by signing the Informed Consent Form (ICF), with one copy being given to the researcher and another to the participant. To ensure confidentiality and privacy regarding the data collected, participants were identified by the letters P (Physicians) and N (Nurses), followed by an Arabic numeral (P1, P2, P3,…, P8/N1, N2,… N11), according to the interviews conducted, to illustrate the description of statements about the topics established in data analysis.

### Theoretical-methodological framework and study design

This is a phenomenological study based on Max Scheler’s theory of values^(14)^, focusing on the comprehensive dimension of value. The philosopher argues that values exist *a priori*, i.e., they are universal and independent of the subjectivity of those who recognize them. These values are understood through healthcare professionals’ practices and actions^(14)^. The experience of being in the world, expressed here in the care provided to women undergoing abortion, allows for ascension in a hierarchy of values organized into ascending categories: useful values, vital values, and spiritual values, which encompass aesthetic, moral, ethical, intellectual (logical), and religious values^(14)^. The study emphasizes the values present in obstetric care in abortion contexts, aligning itself with a model of humanization that promotes support for women’s lives and professional ethics.

Regarding methodological rigor, the COnsolidated criteria for REporting Qualitative research (COREQ) was used, which served as a basis for preparing the report of this qualitative research^(18)^.

### Participants and data sources

Nineteen healthcare professionals participated in the study, including 11 nurses and eight physicians, who work in abortion care in the Metropolitan Region of the state of Pará, Brazil.

The recruitment technique called snowball sampling^(19)^ was used, which consists of a non-probabilistic technique that does not fix the number of subjects in the sample. In this type of sampling, structured by means of the first seed, directed by the researcher, based on knowledge of her work at the reference hospital for legal abortion in Belém, six more professionals were indicated: three physicians and three nurses working in abortion care, facilitating the interviewer’s rapport with her target audience^(19)^.

During this process, each indicated participant was approached and invited via WhatsApp^®^ application, and upon a positive response, the main researcher applied the following eligibility criteria: nurses and physicians working in the care of women undergoing abortion. The exclusion criteria considered professionals who were performing management functions. Thus, data collection was scheduled to take place remotely via Google Meet^®^.

At the time of the initial approach, an invitation was made to each indicated contact and information was provided about the study, such as topic, objectives, type of data collection techniques, analysis, risks and benefits. For those who accepted the invitation, the interview was scheduled on a day and time established by participants.

There was only this initial contact before data collection, to make the invitation and provide explanations about the study. There were no refusals from participants; only a longer time for data collection, given the time available to participants, following COREQ recommendations^(18)^.

### Data collection and organization

Data collection was carried out by conducting a semi-structured interview individually and remotely via Google Meet^®^ between February and May 2023. Conducting the interview as a form of data collection is justified, favoring greater availability of participants in view of their personal and professional commitments, especially in health.

The interview took place with only the main researcher and the interviewee present, through a question asking participants if they were alone at that moment, and, if confirmed, the interview began. Each interview lasted an average of 45 minutes. It occurred in a single moment, answering the interview instrument with open-ended and closed-ended questions.

It should be noted that a pilot study was carried out with three participants, who were not included in the study, to verify the suitability of the interview for the research objectives and nurses’ and physicians’ statements.

The instrument contained questions about gender, age, race, religion, marital status, academic background, year of graduation, specialization, length of professional experience and job tenure. It also had guiding questions such as: how do you perceive the practice of abortion in Brazil? What are the real challenges for women’s health regarding abortion? Statements were recorded using the application’s recording feature, with prior authorization from participants, and later transcribed in full to ensure reliability in the material processing process.

It is worth mentioning that the interviews were conducted only by the main researcher, a female researcher with the following credentials: nurse specialized in nursing-midwifery. She alone collected the data so that there would be no interference from other methods of conducting the interview in this study. It is worth noting that the researcher, at the time, had a course and training in the qualitative research techniques used in the study, and was always monitored after data collection with feedback from her advisor and other team members. They did not interfere with the data collection process; they are research physicians, in addition to having experience in conducting the interview in other studies. Thus, the research team was responsible for processing and analyzing the data.

Data collection was concluded, and the number of study participants was established through the theoretical saturation process^(20)^. This occurs when the data do not provide any new elements to guide or deepen the theorizing and the addition of new information is no longer necessary^(20)^, thus giving a core of meaning to the problem and reaching the saturation criterion. After the 16^th^ interview, three more collections were carried out and, as no other new representative elements were obtained, study data were considered saturated. This is a criterion that allows establishing the validity of a data set, as determined by COREQ^(18)^.

After transcribing the material, the researcher provided feedback on the transcribed material within three days via WhatsApp^®^, ensuring data reliability precepts, as determined by COREQ^(18)^ guidelines, where there were no comments other than what was reported in relation to transcribed material.

### Data analysis

Data analysis was performed based on the content analysis technique^(21)^, aiming to understand participants’ speeches through three chronological stages, namely: pre-analysis of interviews, where material organization took place: transcription of each interview in full; and submission to text skimming and establishment of initial contact with study data, which sought in the first message obtained from the data, with the choice of documents that would compose the study *corpus*, according to data on exhaustiveness, representativeness, homogeneity and relevance^(21)^.

Subsequently, the material exploration stage took place, where coding interventions were constructed. Thus, the word that was established by the colometry technique (Parking in different colors and captions the central topic of each word and phrase of the statements) was verified. The topics in the process can be exemplified as follows: value and women’s right to abortion; value within the scope of legislation; value of life; religious, moral and political values; discomfort in participating in the legal abortion procedure; humanized care and assistance; and reduction of maternal mortality and unsafe abortions.

The purpose of this stage of content analysis seeks the registration unit based on the topic. Thus, registration units were established with the relevance of the rereading of all the material and, thus, the grouping, namely: value and life; religious and moral value; ethics in abortion; non-value of abortion; duality between moral and religious value and the value of life; vital value in the field of health; maternal mortality x vital value^(21)^, which subsidized the formation of three thematic axes and three thematic categories, as expressed in Chart 1.

**Chart 1:** Establishment of registration units, thematic axes and thematic categories of the study

Recording unit	Thematic axis	Thematic category
Value and life	Values ​​of professional practice for women’s lives.	Values ​​attributed by nurses and physicians to the legal abortion process
Religious and moral value		
Ethics in abortion		
Non-value of abortion	The lack of value for abortion.	The lack of value given to abortion in professional practice
Duality between moral and religious value and the value of life		
Vital value in the field of health	The health and vital value of women.	The vital value of unsafe abortion: a public health issue
Maternal mortality x vital value		

In the last chronological stage of the analytical process, treatment of results, inference and interpretation, the constructive elements were classified by the process of mutual exclusion, homogeneity, relevance, objectivity and fidelity, and productivity, aiming at categorization^(21)^. Thus, the thematic axes established in the analytical process allowed formulating the following thematic categories: 1) Values attributed by nurses and physicians to the legal abortion process; 2) The lack of value given to abortion in professional practice; and 3) The vital value of unsafe abortion: a public health issue. The established categories were discussed based on Max Scheler’s theory of values^(14)^ framework and Brazilian public policies regarding reproductive health as well as specialized literature on abortion.

## RESULTS

As for participant characterization in terms of gender, there was a predominance of 15 females and four males. In relation to age, there was a predominance of seven participants over 50 years old, followed by five between 31 and 40 years old, four between 21 and 30 years old and three between 41 and 50 years old.

Regarding self-declared race, the majority were ten brown and nine white. Concerning religion, there was a predominance of 12 Catholics, followed by four Protestants, one Spiritist, one Kardecist and one Afro-descendant. In terms of marital status, there was a predominance of eight married people, followed by six single people, four in a stable union and one divorced person.

In relation to the training institution, 15 participants came from public institutions and four from private institutions. Regarding the year of training, there was a predominance of nine participants with training older than 2015, followed by seven who graduated between 2006 and 2015 and four who graduated between 1995 and 2005.

All had a *lato sensu* specialization in nursing-midwifery and obstetrics and gynecology. With regard to the length of their respective performance as a nurse or physician, there was a predominance of seven participants between 5 and 10 years, followed by six between 11 and 20 years, five with less than 5 years and one with more than 20 years of experience in obstetrics/nursing-midwifery.

Regarding job tenure, there was a predominance of nine with less than 5 years, followed by five with more than 20 years, three with between 11 and 20 years, and two with between 5 and 10 years of service.

The following data illustrate the breakdown of values attributed to the analytical process of the study, with its methodological consistency, which is presented below:

### Values attributed by nurses and physicians to the legal abortion process

The values attributed by healthcare professionals show that legal abortion constitutes a woman’s right, especially a vital value inherent to life, which must be respected in situations covered by Brazilian legislation, guaranteeing Brazilian women’s lives, as highlighted in the following statements:

*It is a woman’s right in cases of rape of girls under 14 years old, like any other woman. In cases of maternal risk or anencephaly, we are still far behind, because there are other cases of fetal malformations incompatible with life that are not included.* (N1)

*It means a right to life, a right that a person has to not be just an incubator, to not be seen as just an object that carries a life inside. There are two lives, so for me, it means looking at the woman as she is, being a life too, and looking at the situation as two lives and not one life in an object.* (P4)

Healthcare professionals’ statements demonstrate their perceptions about the discussions about abortion, whether in the political sphere, in health units, or between managers and professionals. These result in a care model that is influenced by moral and ethical values, belonging to taboos supported by dogmatic issues with conservative agendas, which are values that are contrary to the lives of women who need abortion as provided for by law, as shown in the following excerpts:

*Patients who seek legal support are able to have an abortion, but there are still some obstacles in terms of care. Some professionals do not agree to perform the procedure and postpone the procedure for religious and moral reasons, and do not participate in post-abortion curettage.* (N4)

*Today, we are seeing mortality in the country due to delayed care. Neglect of care is reflected in social, economic and racial inequality, because women with money have better quality care, and when we work in the SUS, we realize how much these social inequalities are marked in women in terms of gender violence, racial and gender discrimination, transgender men having abortions, religious intolerance and other things that will be reflected in violence in care for women.* (P2)

### The lack of value given to abortion in professional practice

It is clear that professionals make their decisions influenced by several value factors, such as principles of professional ethics, religion, political issues, understanding of abortion legislation and the need to accommodate individuals in vulnerable situations. Sometimes, this analysis is limited to compliance with what is established in case law. However, many healthcare professionals show discomfort in taking a position on the topic, treating it as a controversial subject, as evidenced in the following statements:

*Abortion is necessary as long as the law is followed; we have to follow the rules. As a Christian, I think that every Christian is against abortion, right? Looking at it from a religious perspective, but as a professional, we have to follow what is regulated by law. I am in favor, as long as it really does meet the criteria established by law.* (N2)

*I am against abortion. It is a subject that is being discussed a lot right now, with many things wanting to be changed, very radical changes. I think that what is being proposed today is something that should be discussed, and some limits should be considered, checking special situations that need more details to be allowed. I think that a lot of discussion is still needed. But I am against abortion, allowing all types of abortion.* (P3)

Concerning the professionals who reported not participating, they did not value abortion and the care for women’s own health. Due to their discomfort with the subject, the influence of religious values and dualistic thinking between life and death were perceived, as well as the professionals who stated that they were totally against participating in the process of terminating pregnancy for legal reasons, emphasizing greater discomfort in cases resulting from sexual violence, as per the following statements:

*The physician is not being forced to do it. We can refuse to do it for personal or religious reasons, and I would put myself in that position. I do not agree, and only in situations where the mother’s life is at risk, and there are very few reasons why we would have to save the mother’s life by performing an abortion; that is the only reason I would do it. But even so, as a last resort, if there was no one to do it in my place, if it was an imminent risk, well, I would not do it.* (P6)

*I would participate, depending on the case. In some cases, I wouldn’t feel comfortable, it’s a matter of faith, but in most of them I would participate, thinking about the women. For instance, a woman with a heart problem and for whom pregnancy is a risk for her, or a woman who has a malformed baby that is incompatible with life and who really would not have any advantage in carrying the pregnancy to term and who wants it. In other cases, I would prefer not to participate, such as cases in which the mother is well, the baby is well, and the baby could be adopted if this person were well received and referred for adoption. If it were possible, I would refer the baby.* (P4)

### The vital value of unsafe abortion: a public health issue

Healthcare professionals associate unsafe abortions with a greater chance of negative repercussions on women’s health, such as preventable maternal deaths, in addition to the impact on women’s bodies. Vital values are inherent to women’s health, since an unsafe practice can result in death, as shown by its negative outcomes:

*Unsafe abortion is one of the main causes of death among women; unfortunately, it can cause psychological distress and ineffective access to rights, public policies, and the women’s healthcare network. So, when unsafe abortion happens, I think it is triggered by a series of factors: prejudice, social judgment, and the population’s lack of health education.* (N7)

*Morbidity and mortality, near miss, hemorrhage, ICU admission, social problems, lynchings, media exposure, viralization. There is a risk of infection and development of sepsis, a risk of perforation, risk of regret, I think this is the biggest one, because she doesn’t have much choice in making decisions, so she may rely on some illegal position.* (P4)

Professionals perpetuate the seriousness and negative outcomes that unsafe abortions entail. Thus, when considering the decriminalization, legalization and expansion of measures for access to legal abortion in the country, especially in reducing maternal mortality, their worldview establishes a vital value that, in turn, establishes itself as a protector against maternal mortality due to unsafe abortion factors. This occurs with due access to reproductive healthcare services, as per the following statements:

*I am in favor of expanding and facilitating the legal forms of abortion. Women should not be forced to continue a pregnancy; they have the right to make the decision whether they want to or not. Expanding policies for access to legal abortion is very important for women’s health. If they do not have it legally, they will end up having an unsafe abortion.* (N4)

*If we look at countries that have managed to do this, we know more or less what will happen: a decrease in maternal mortality caused by infection, hemorrhage and unsafe abortions. We see greater adherence to reproductive planning, and I believe these are the two main things: a reduction in maternal mortality and an expansion of reproductive planning services.* (P2)

In this way, access to legal abortion becomes a vital value and a centrality in women’s lives, and its decriminalization constitutes an important practical reflection as a milestone for guaranteeing sexual, reproductive and human rights in women’s lives.

## DISCUSSION

Healthcare professionals recognize that legal abortion constitutes reproductive justice^(12)^ and a vital value^(14)^ for women, and should be guaranteed by the Brazilian State in legal provisions. However, the possession of the value for women’s lives and recognition by healthcare professionals do not make it effective in practice, making it necessary to raise awareness of reproductive justice in order to implement legal abortion. Hence, there is inertia in discussions between professionals and managers of healthcare services, as well as the lack of information for women about their rights, especially in legislation, which guarantees abortion resulting from sexual violence, maternal risk and/or fetuses with anencephaly^(7)^. What is needed is the implementation of the vital value of abortion and, if the State respects this practice, women’s lives will have a sense of guaranteed value^(14)^.

The essence of the description of the phenomena is based on emotional intuition, in which the vital value is understood by emotion with a sense of affective value. This is a vital factor that has value for women’s lives within the scope of the well-being of women in situations of abortion. Thus, the focus of the study’s discussion based on the philosopher discusses the relationship between vital value^(14)^ and healthcare, emphasizing the importance of women’s well-being, safety and life. According to the axiological perspective, this relationship is fundamental to healthcare professionals’ performance^(14)^. Furthermore, it highlights that any factor that opposes these issues represents a threat to women’s lives.

In this regard, in Uruguay, for instance, even after abortion was legalized, women who sought legal abortion services in the public sector reported feeling judged by healthcare professionals. These results highlight the importance of raising awareness and training health teams and specialized services with humanization, empathy and attentive listening, which are crucial strategies to reduce discriminatory behavior towards women seeking abortion care^(22)^. Despite the legalization of abortion in Uruguay, discriminatory acts against women still occur. This perpetuates the idea that women’s lives are not considered as vital or valuable by healthcare professionals.

Since it is a recurring issue in the media, abortion is linked to traumatic situations experienced by women as a result of intimidation, coercion and violence. This creates obstacles to terminating a pregnancy, even when legally protected. These factors accentuate the marks of moral judgment and prejudice, which are strongly present in healthcare professionals’ discourse. These discourses end up translating into inappropriate attitudes in their practice of caring for women^(1,2,4,7,8)^.

The Brazilian National Abortion Survey reveals the recurrence of abortion among women of different age groups, racial groups, educational and social levels and religious beliefs. This situation highlights vulnerabilities and inequalities in access to quality healthcare services, especially for black, brown and indigenous women, and those with low income and education levels. These are the cases that frequently face maternal deaths resulting from unsafe and clandestine abortions^(23)^. Racial and class inequality puts women in a vulnerable situation when it comes to reproductive justice, reflecting the reality of inequalities and the value of rich women’s lives, who pay for the procedure, while poor and black women die^(24)^.

The dimensions contrary to legal guarantees that go beyond the ethical parameters of the profession become real obstacles constantly experienced by Brazilian women. They seek legal abortions, despite professional issues, conscientious objection from physicians, refusal by the multidisciplinary team, distrust of their claims, judgment and prejudice. The main barrier mentioned by black women was fear of mistreatment, especially those who declared having induced the abortion. In addition, they face obstacles in accessing healthcare services, which are mostly concentrated in large urban centers in the country, and pilgrimages to health units. These factors influence them to give up their rights and encourage the search for unsafe and clandestine abortions^(23,25)^.

A study^(26)^ states that abortion care is an essential component of comprehensive reproductive justice care for women’s healthcare, as mentioned by the American College of Obstetricians and Gynecologists. Thus, the relationship between healthcare professionals and women who seek the service to exercise their right must be based on respect and information. Moreover, they have an obligation and an ethical value in providing abortion care, which tends to include providing information, abortion management, and post-abortion care, which protects women’s dignity and bodily autonomy.

Discrimination in healthcare services is a recurring problem for women who face abortion situations, manifesting itself directly and indirectly. This includes inhumane treatment, moral judgment and embarrassment, which often manifests itself as forms of violence when these women are treated. This lack of dignity in hospital care ends up affecting even women who have experienced spontaneous abortions, who are unjustly suspected of having induced the abortion^(27)^.

Currently, we are witnessing a growing conservative movement that opposes women’s rights, reflecting a devaluation of the lives and safety of women in situations of abortion. According to the theory of values, healthcare professionals guide their actions based on principles that seek to satisfy their emotional needs and intuitions, promoting a sense of fulfillment in their work practice^(14)^. When physicians and nurses who provide abortion care base their judgments on moral or religious issues, they penalize women’s lives, treating them as if their lives were of less value. This occurs even in circumstances where abortion is warranted, such as in cases where women’s lives are at risk, pregnancy resulting from rape, or fetus anencephaly.

This context generates not only discrimination and judgment by society and healthcare professionals, but also increasing pressure to penalize these women for simply exercising their rights. The moral and ethical value that relates to human beings’ spiritual aspect is fundamental and presents itself as a phenomenon intrinsic to each person^(14)^.

These values are deeply connected to woman care and human life well-being, requiring an ethical approach that respects the dignity of others. The spiritual value that involves women’s lives transcends purely rational conduct, and is referred to by the philosopher as a “sympathetic conduct” - an ethical care towards others. Sympathizing implies a shared experience that goes beyond ephemeral emotions, manifesting itself as an act of love for the other^(28)^.

Thus, recognizing women as the ethical foundation that carries intrinsic value, we must strive to genuinely care for human life. This implies perceiving others’ experiences - their pain and struggle - with a comprehensive vision of the human being as a spiritual individual^(28)^.

A study^(29)^ found that professionals have an ethical duty, in particular, to hold States accountable for their obligation to respect, protect and fulfil the right to life and the right to health, especially to take measures to reduce maternal mortality and morbidity. Furthermore, they must ensure that, where abortion is legal, it is safe and accessible.

The Brazilian Federation of Gynecology and Obstetrics Associations^(30)^ recommended that physicians make every effort to perform legally permitted abortions in cases of pregnancies resulting from sexual violence, prioritizing the early stages of pregnancy and, especially, before 22 weeks of gestation. It is essential that physicians act impartially, welcomingly, without passing judgment, and provide pregnant women with all the information about their rights. If it is not possible to perform the procedure on site, pregnant women should be promptly referred to another service that has the necessary conditions to perform the procedure. However, in the recent context, in 2024, there was a presentation of Bill 1904/2024, with great national repercussion, which provides that abortion performed after 22 weeks of gestation, in any situation, will be considered homicide, including in cases supported by Brazilian legislation for abortion^(31)^, in which there is an unveiling of a professional practice that has a countervalue and no value for the issues inherent to abortion.

At this point, despite considering abortion in accordance with the law, professional practice has revealed discomfort and non-acceptance on the part of some physicians and nurses in performing legal abortions due to issues related to the duality between life and death associated with religion. Thus, there were more notable cases of pregnancies resulting from sexual violence, with coercion and the idea of guiding women to continue their pregnancy and later send their baby for adoption. This movement distances itself from the guidelines of welcoming and guiding with impartiality. Thus, it is up to healthcare professionals to adopt a therapeutic attitude, and it is strictly forbidden to impose their values during woman welcoming and when providing guidance on abortion. Nor should undue emphasis be placed on supposed risks that do not correspond to reality in an attempt to coerce women to rethink their decision^(4)^.

When there is this phenomenological unveiling through the act of counter-valuation and non-value by professionals, who are against any issue arising from abortion or do not have a formed position on the subject, it results in a counter-value and non-value to life and to the human person, which in this case is configured in women^(14)^.

A healthcare professional who does not support a woman’s choice to have an abortion violates the profession’s core ethical obligations, such as autonomy, beneficence, nonmaleficence, and justice. The obligation to respect and promote women’s autonomy, the linchpin of modern medical ethics, is a central justification for the broad ethical duty of clinicians to provide health information^(26)^. Therefore, physicians and nurses have an obligation to guarantee information about abortion and respect women’s choice, considering their lives as the primary value to be ensured.

According to WHO statistics^(4)^, approximately 25 million unsafe abortions are performed worldwide each year. In areas where the practice is legal, the rate of unsafe terminations is around 10%. However, in regions where abortion is criminalized, this number jumps to 25%. It is alarming to estimate that approximately 39,000 women are hospitalized or die each year due to complications resulting from unsafe abortions. It is crucial to emphasize that the vast majority of these tragedies could have been avoided if they had been performed by qualified professionals and according to recommended methods. Unsafe and clandestine abortions not only represent a serious public health concern, but are also emerging as one of the leading causes of maternal mortality worldwide, as highlighted by the WHO^(32)^.

Public health is a way of protecting women’s health, and in this sense, it has a vital value, the essence of which is life for the human person. Thus, criminalization brings with it substantial risks to women’s mental health. These risks arise from unplanned pregnancies, which can lead to terminations through unsafe abortions, contributing to their death and the lack of appreciation of their person as an essential part of life. In addition to this, there is a greater propensity to develop postpartum depression and adopt less healthy behaviors during pregnancy. These behaviors can lead to complications that even affect future children^(33)^.

An example of equity and reproductive justice, in Canada, abortion is recognized and regulated as a healthcare service. The legality of abortion services is not subject to changes in the political climate or changes in social attitudes. Thus, politicizing the issue misrepresents the aspect of health equity and, in this country, there is a deep respect for personal autonomy and medical integrity, ensuring that abortion, as a healthcare service, remains accessible and adaptable^(34)^. Thus, Brazil has much to learn and has this initiative as an example for Brazilian women and healthcare professionals’ practice.

Concerning the decriminalization of abortion as a path that enables comprehensive and safe care for women, professionals demonstrate agreement and positive perspectives regarding the reduction of maternal mortality and expansion of access to reproductive healthcare services. Countries that have less restrictive abortion laws have shown a reduction in maternal mortality, expansion of contraception, opportunities for counseling and increasing insertion of long-acting methods, such as the IUD, after the procedure to terminate pregnancy, which are well accepted by women. These results demonstrate the favorable impacts of legalizing abortion, ensuring greater accessibility in the healthcare system^(24,35,36)^.

Recent guidelines presented by the WHO^(4)^ in the Safe Abortion Guide highlight the delay and slowness with which Brazil is progressing towards the decriminalization of abortion and the expansion of access to healthcare services. Meanwhile, a wave of legalization of abortion is gaining momentum in neighboring countries.

Thus, accessibility to legal abortion and its decriminalization represent a crucial milestone in ensuring reproductive justice with social equity for women. The fight for progress on these issues in Brazil is extremely urgent, involving not only society in general, but also healthcare services, their administrators and the professionals involved. Providing information, making specialized services available and promoting reproductive planning are essential strategies for reducing the number of unsafe abortions in the country and maternal mortality. It is imperative that there be sexual education as a means of decision-making, adoption of contraceptive methods to avoid abortion and availability of safe abortion to prevent deaths.

Thus, there is a need to guarantee women’s health as a matter of value. When healthcare professionals express themselves in favor of sexual and reproductive rights, this allows them, through their emotional perception, to guarantee a vital value, a moral and ethical value for the care of the human person and, in this case, the woman free from any harm resulting from an unsafe act.

### Study limitations

The study was limited by the use of other collection techniques, such as observation, because the interview took place online.

### Contributions to nursing, health or public policy

The study contributes to knowledge about the values attributed by physicians and midwives to legal abortion, through facts that today permeate the vital (for life), ethical-professional and moral values of women. These values are fundamental in their professional performance, in the sense of a worldview, which directly impacts their practice.

## FINAL CONSIDERATIONS

This study aimed to understand the values attributed by nurses and physicians working in obstetric care in relation to abortion. The results revealed a discourse that encompasses vital, moral and ethical aspects in the context of women’s health. For healthcare professionals, patients’ health and physical well-being are fundamental values, which directly influence their care practice.

On the other hand, there are also counter-values and a lack of values in philosophical education on the subject. Abortion is often seen as an indication of a lack of an affirmative position, directly impacting women’s lives, especially in relation to maternal mortality.

In a secular state like Brazil, the discussion on abortion - considered a serious public health problem when performed unsafely - should not be guided by religious and moral issues, since this redirects the debate in inappropriate directions. It is imperative to guarantee reproductive justice and social equity, promoting the inclusion of this issue in public policies. Abortion should be seen as a strategy to value women’s lives and combat maternal mortality.

This dynamics highlights the moral and ethical dilemmas faced by healthcare professionals regarding the legal termination of pregnancy. While some recognize the importance of offering care based on acceptance and humanization, others find justifications for abstaining from this role. Often, these professionals’ practice does not prioritize women’s rights, but rather a context of judgment, coercion, and discrimination regarding their decisions about legal abortion.

Therefore, the decriminalization of abortion in Brazil becomes a crucial topic for research, requiring the support of healthcare professionals, managers, politicians and society as a whole, since unsafe abortion in the country contributes to high rates of maternal mortality. Therefore, studies that investigate both legal abortion, its decriminalization and its legalization are essential for a broader understanding of this issue.

## References

[1] Ministério da Saúde (BR) (2011). Atenção humanizada ao abortamento: norma técnica.

[2] Galli B. (2020). Challenges and opportunities for access to legal and safe abortion in Latin America based on the scenarios in Brazil, Argentina, and Uruguay. Cad Saúde Pública.

[3] Morais LR. (2008). A legislação sobre o aborto e seu impacto na saúde da mulher. Senatus.

[4] World Health Organization (WHO) (2022). Abortion care guideline.

[5] Cardoso BB, Vieira FMSB, Saraceni V. (2020). Aborto no Brasil: o que dizem os dados oficiais?. Cad Saúde Pública.

[6] Acayaba C, Figueiredo P. (2020). SUS fez 80,9 mil procedimentos após abortos malsucedidos e 1.024 interrupções de gravidez previstas em lei no 1º semestre de 2020.

[7] Domingues RMSM, Fonseca SC, Leal MC, Aquino EML, Menezes GMS. (2020). Unsafe abortion in Brazil: a systematic review of the scientific production, 2008-2018. Cad Saúde Pública.

[8] Machin R, Couto MT, Rocha ALS, Costa MRM. (2019). Formação médica e assistência aos processos de abortamento: a perspectiva de residentes de duas universidades públicas em São Paulo, Brasil. Interface (Botucatu).

[9] Organização Mundial de Saúde (OMS) (2011). Avaliação da qualidade do cuidado nas complicações graves da gestação: a abordagem do near miss da OMS para a saúde materna.

[10] Ministério da Saúde (BR) (2021). Boletim Epidemiológico 29: mortalidade proporcional por grupos de causas em mulheres no Brasil em 2010 e 2019.

[11] Menezes GMS, Aquino EML, Fonseca SC, Domingues RMSM. (2020). Abortion and health in Brazil: challenges to research within a context of illegality. Cad Saúde Pública.

[12] Brandão ER, Cabral CS. (2021). Justiça reprodutiva e gênero: desafios teórico-políticos acirrados pela pandemia de Covid-19 no Brasil. Interface (Botucatu).

[13] Dantas RLS, Melo MIB, Pinto LMTR, Guimarães ACM, Ramos KS. (2019). Concepção dos profissionais de saúde acerca do aborto previsto em lei. Enferm Foco.

[14] Scheler M. (2012). Da reviravolta dos valores.

[15] Goes EF, Menezes GMS, Almeida MCC, Araújo TVB, Alves SV, Alves MTSSB (2020). Racial vulnerability and individual barriers for Brazilian women seeking first care following abortion. Cad Saúde Pública.

[16] Testoni I, Finco N, Keisari S, Orkibi H, Azoulay B. (2021). Conflicts between women’s religiosity and sense of free will in the context of elective abortion: a qualitative study in the worst period of Italy’s COVID-19 Crisis. Front Psychiatry.

[17] Wolfe T, Rodgers YVM. (2022). Abortion during the COVID-19 Pandemic: racial disparities and barriers to care in the USA. Sex Res Social Pol.

[18] Tong A, Sainsbury P, Craig J. (2007). Consolidated criteria for reporting qualitative research (COREQ): a 32-item checklist for interviews and focus groups. Int J Qual Health Care.

[19] Vinuto J. (2014). A amostragem em bola de neve na pesquisa qualitativa: um debate em aberto. Temáticas.

[20] Fontanella BJB, Luchesi BM, Saidel MGB, Ricas J, Turato ER, Melo DG. (2011). Amostragem em pesquisas qualitativas: proposta de procedimentos para constatar saturação teórica. Cad Saúde Pública.

[21] Bardin L. (2011). Análise de conteúdo.

[22] Makleff S, Labandera A, Chiribao F, Friedman J, Cardenas R, As E (2019). Experience obtaining legal abortion in Uruguay: knowledge, attitudes, and stigma among abortion clients. BMC Women’s Health.

[23] Diniz D, Medeiros M, Madeiro A. (2017). National Abortion Survey 2016. Ciênc Saúde Colet.

[24] Batalha E. (2018). Hora de enfrentar o tabu. RADIS.

[25] Rocha WB, Silva AC, Leite SML, Cunha T. (2015). The perception of health professionals regarding legal abortion. Rev Bioét.

[26] Oberman M, Lehmann LS. (2023). Doctors’ duty to provide abortion information. J Law Biosci.

[27] Madeiro AP, Rufino AC. (2017). Maus-tratos e discriminação na assistência ao aborto provocado: a percepção das mulheres em Teresina, Piauí, Brasil. Ciênc Saúde Colet.

[28] Alves VH, Barea R, Werneck VR, Grzibowsk S, Rodrigues DP, Silva LA. (2018). Ethical care of the other: Edith Stein and Max Scheler’s contributions. Esc Anna Nery.

[29] Londras F, Cleeve A, Rodriguez MI, Farrell A, Furgalska M, Lavelanet AF. (2022). The impact of provider restrictions on abortion-related outcomes: a synthesis of legal and health evidence. Reprod Health.

[30] Federação Brasileira das Associações de Ginecologia e Obstetrícia (Febrasgo) (2022). Recomendação oficial FEBRASGO junto ao CFM acerca de idade gestacional na interrupção de gravidez.

[31] Agência Brasil (2024). Manifestantes vão às ruas contra a PL que equipara o aborto ao homicídio.

[32] Organização Mundial da Saúde (OMS) (2013). Abortamento seguro: orientação técnica e de políticas para sistemas de saúde.

[33] Anis Instituto de Bioética (2019). argumentos apresentados ao Supremo Tribunal Federal na Audiência Pública da ADPF 442.

[34] Timbo CZ, Rodriguez A. (2023). Decriminalizing Abortion: a journey towards access and equity.

[35] Raymond E, David G. (2012). The comparative safety of legal induced abortion and childbirth in the United States. Obstet Gynecol.

[36] Latt SM, Milner A, Kavanagh A. (2019). Abortion laws reform may reduce maternal mortality: an ecological study in 162 countries. BMC Women’s Health.

