# Supplementary material for: The values attributed by physicians and nurses to abortion in Brazil
Source: Rev Bras Enferm. 2025 Mar 31;78(2):e20240196. doi: 10.1590/0034-7167-2024-0196 (PMC11961044; doi:10.1590/0034-7167-2024-0196)
Supplement: Translation

# Translation: Os valores atribuídos por médicos e enfermeiros ao abortamento no âmbito do Brasil

**DOI:** 10.1590/0034-7167-2024-0196pt

**Authors:** Ana Zélia Silva Fernandes de Sousa, Valdecyr Herdy Alves, Andressa Tavares Parente, Bianca Dargam Gomes Vieira, Audrey Vidal Pereira, Lucia Helena Garcia Penna, Giovanna Rosario Soanno Marchiori, Diego Pereira Rodrigues

**Affiliations:** I Universidade do Estado do Pará. Tucuruí, Pará, Brasil; II Universidade Federal Fluminense. Niterói, Rio de Janeiro, Brasil; III Universidade Federal do Pará. Belém, Pará, Brasil; IV Universidade do Estado do Rio de Janeiro. Rio de Janeiro, Rio de Janeiro, Brasil; V Universidade Federal de Roraima. Roraima, Boa Vista, Brasil

**Keywords:** Mulheres, Obstetrícia, Aborto Legal, Pessoal de Saúde, Direitos Sexuais e Reprodutivos.

## Abstract

**Objetivos:**

compreender os valores atribuídos pelos enfermeiros e médicos atuantes na assistência obstétrica ao abortamento.

**Métodos:**

trata-se de estudo fenomenológico fundamentado no referencial da teoria dos valores de Max Scheler, com 19 entrevistas semiestruturadas *online* com profissionais de saúde atuantes no abortamento legal, por meio da técnica de recrutamento *snowball sampling*. Os dados coletados foram transcritos na íntegra e submetidos à análise de conteúdo.

**Resultados:**

evidenciou-se, pelos depoimentos dos profissionais de saúde, que o abortamento possui um valor vital, moral e ético profissional. Além disso, mostrou-se o desvalor do abortamento por parte de médicos e enfermeiros, e ainda o valor vital para a vida das mulheres atribuído pela saúde pública.

**Considerações Finais:**

com a finalidade da compreensão dos valores atribuídos por enfermeiros e médicos, mostrou-se a necessidade do avanço para os valores à saúde, ao direito, à dignidade e ao respeito das mulheres diante do abortamento.

## INTRODUÇÃO

As mortes maternas decorrentes de abortamento no Brasil ocupam uma posição em destaque no cenário obstétrico. As consequências dos abortamentos são notadas principalmente em mulheres em condições socioeconômicas mais vulneráveis. A literatura especializada aponta que, nos países em desenvolvimento, como os da América Latina, Ásia e África, obtém-se um risco de morte por abortamento inseguro, em comparação aos realizados em países desenvolvidos. Países desenvolvidos, como Estados Unidos da América, França e Alemanha, apresentam métodos mais modernos, bem como uma legislação que apoia o abortamento sob um aspecto não punitivista. Assim, o avanço dos aspectos jurídicos e legais para o aborto legal se torna um valor à discussão atual para a sociedade brasileira^(1-8)^. De acordo com levantamento da Organização Mundial da Saúde (OMS), apenas a metade dos abortos registrados no mundo é realizada por método recomendado^(4)^.

A OMS^(9)^ estima que ocorram a cada ano 22 milhões de abortamentos inseguros no mundo, ocasionando cerca de 47 mil mortes de mulheres. Com base nos dados disponibilizados no site do Departamento de Informática do Sistema Único de Saúde (SUS), no primeiro semestre de 2020, o número de procedimentos realizados no SUS decorrente de complicações após abortamentos superou em 79 vezes as interrupções gestacionais previstas em Lei^(2,10)^, mostrando ser um problema de saúde pública.

Estudo realizou um levantamento pelo Sistema de Informações sobre Nascidos Vivos e pelo Sistema de Informação sobre Mortalidade durante o período de 2006 a 2015. Foram encontrados 770 óbitos maternos com causa básica de aborto no Sistema de Informação sobre Mortalidade. O aborto não especificado se manteve como a causa básica mais frequente entre os óbitos por aborto no período avaliado, com média de 56,5% dos casos. Entre os 770 óbitos com causa básica declarada como aborto, apenas 0,9% dos óbitos foram devido ao aborto por razões médicas e legais; 14,9% foram declarados como abortos espontâneos; 15,2% foram declarados como outros tipos de aborto; e 12,5% foram declarados como falha de tentativa de aborto^(5)^.

No Brasil, o abortamento é crime previsto pelo Código Penal nos Artigos 124, 125 e 126, com penalidades para a mulher e para o médico que o praticam, entretanto, desde 1940, na jurisprudência brasileira, prevê-se o abortamento legalizado para: 1) gestações resultantes de estupro (precedido de consentimento da gestante ou, quando incapaz, de seu representante legal); 2) em casos em que não há outro meio de salvar a vida da gestante, pelo seu risco de morte; e 3) recentemente, em 2012, por meio da Arguição de Descumprimento de Preceito Fundamental (ADPF) n^o^ 54, o Supremo Tribunal Federal (STF) adicionou a gravidez de feto com anencefalia como condição para interrupção da gestação, fornecendo essas alternativas legais para a sua autorização^(7,11)^.

Nesse sentido, o aborto legal ou seguro, que constitui a interrupção da gestação permitida por lei, deve ser exercido apenas por profissionais qualificados, com suporte de regulamentações, políticas públicas e infraestrutura adequada dos sistemas de saúde, fornecendo suprimentos, equipamentos e fluxos de atendimento para a mulher ter acessibilidade a esses serviços. A não efetivação de condições favoráveis representa um atentado à vida e à saúde das mulheres brasileiras, necessitando de uma justiça reprodutiva mediante o descaso do poder público em assegurar o seu direito como valor^(1,3,12)^.

O abortamento previsto em lei é visto pelos profissionais de saúde como um direito conquistado pelas mulheres, apesar de alguns (des)conhecerem a legislação e se manterem afastados nesse processo, devido ao seu posicionamento moral, religioso ou ideológico contrário. Nos serviços de saúde onde se disponibiliza o abortamento legal, nota-se por parte dos profissionais a insuficiência na qualificação sobre a temática e desalinhamento com as normas técnicas do Ministério da Saúde, evidenciando tal lacuna para a atuação desses^(13)^.

Assim, em 2022, a OMS^(4)^ apresentou diretrizes atualizadas sobre a proteção da saúde de mulheres e meninas com relação à realização de abortamentos, objetivando apresentar recomendações e melhores práticas acerca do procedimento, visando possibilitar assistência global de qualidade baseada em evidências. Entre as diretrizes, destacam-se a importância do fortalecimento na disseminação de informações sobre métodos contraceptivos, a urgência em se retirar as barreiras políticas desnecessárias existentes, como a criminalização, período de espera obrigatório, anuência de familiares ou mesmo outras formas de impedir ou retardar o procedimento, descriminalizando o abortamento e tornando-o acessível às meninas e mulheres.

A compreensão dos valores dos profissionais de saúde que atuam na assistência obstétrica é fundamental para promover práticas que garantam a realização do abortamento sem desrespeitar a mulher. É essencial que se busque uma justiça reprodutiva em relação ao abortamento, ampliando os direitos humanos e perseguindo a equidade social^(12)^. A realidade do trabalho na realização de abortamentos legais deve implicar uma transformação na assistência prestada ao país, destacando a importância do cuidado efetivo com a mulher, considerando seus sentimentos, afetividades e a subjetividade decorrente da situação. Isso inclui a defesa dos direitos sexuais, reprodutivos e humanos, evitando a negligência do Estado em relação ao abortamento.

Nesse contexto, é necessário, segundo a perspectiva scheleriana, refletir sobre a concepção de valor. Para o filósofo, tudo que possui relevância para a vida de uma pessoa é considerado valor. Cada valor é construído pelo sujeito e visa atender às suas carências, ou seja, um valor se torna significativo quando contribui para a realização das necessidades vivenciais e existenciais da pessoa^(14)^. Outros conceitos presentes na teoria dos valores referem-se ao contravalor e ao não valor; o primeiro se opõe ao valor, enquanto que o segundo expressa indiferença em relação a ele.

Assim, os valores dos profissionais de saúde em relação ao abortamento podem variar. Alguns atribuem um valor positivo à vida e à ética, enquanto que outros manifestam contravalores ou indiferença em relação ao abortamento^(14)^. Ao cuidar de mulheres em situação de abortamento, é fundamental que os profissionais estejam atentos à vida das mulheres, considerando-a um valor essencial que deve ser pautado pelo respeito, dignidade e justiça social e reprodutiva.

Neste sentido, o estudo teve como questão norteadora: quais são os valores que enfermeiros e médicos atuantes na assistência obstétrica atribuem ao abortamento?

Dessa forma, a capacitação de profissionais de saúde na assistência às mulheres em situação de abortamento e o acesso à interrupção prevista na lei brasileira, com oferta de serviços especializados, tornam-se essenciais para a garantia dos preceitos do SUS e dos valores e direitos das mulheres brasileiras. A literatura científica mostra o conflito moral, ético, religioso, jurídico e cultural no abortamento legal, e, no campo da prática profissional, torna-se relevante trazer à luz a problemática da inação diante da concretização dos direitos assegurados às mulheres. Desse modo, esses impactos refletem na imparcialidade na assistência prestada, carregada pelos profissionais de saúde de julgamento, preconceitos e descrença nos relatos apresentados, fragilizando a qualidade e a segurança do cuidado prestado às mulheres que buscam a interrupção legal da gestação^(15-17)^.

## OBJETIVOS

Compreender os valores atribuídos pelos enfermeiros e médicos atuantes na assistência obstétrica ao abortamento.

## MÉTODOS

### Aspectos éticos

O estudo foi conduzido conforme as diretrizes de ética nacionais e internacionais, e aprovado pelo Comitê de Ética em Pesquisa do Instituto de Ciências da Saúde (ICS) da Universidade Federal do Pará, como disposto na Resolução do Conselho Nacional de Saúde (CNS) nº 466/2012.

Foi garantida a participação voluntária mediante a assinatura do Termo de Consentimento Livre e Esclarecido (TCLE), sendo entregue uma via ao pesquisador e outra ao participante. Para assegurar o sigilo e privacidade quanto aos dados que foram coletados, os participantes foram identificados pelas letras M (Médicos) e E (Enfermeiros), seguidas de um algarismo arábico (M1, M2, M3,…, M8/ E1, E2,… E11), conforme a realização das entrevistas, para ilustrar a descrição dos depoimentos acerca dos temas estabelecidos na análise de dados.

### Referencial teórico-metodológico e tipo de estudo

Trata-se de pesquisa fenomenológica fundamentada na teoria dos valores de Max Scheler^(14)^, focando na dimensão compreensiva do valor. O filósofo argumenta que os valores existem *a priori*, ou seja, são universais e independentes da subjetividade de quem os reconhece. Esses valores são compreendidos por meio das práticas e ações dos profissionais de saúde^(14)^. A experiência de estar no mundo, expressa aqui no cuidado prestado às mulheres em situação de abortamento, permite a ascensão em uma hierarquia de valores organizada em categorias de ordem crescente: valores úteis, valores vitais e valores espirituais, os quais englobam valores estéticos, morais, éticos, intelectuais (lógicos) e religiosos^(14)^. O estudo enfatiza os valores presentes na assistência obstétrica em contextos de abortamento, alinhando-se a um modelo de humanização que promove o suporte à vida das mulheres e à ética profissional.

No que diz respeito ao rigor metodológico, foi utilizado o instrumento *COnsolidated criteria for REporting Qualitative research* (COREQ), que serviu como base para a elaboração do relatório desta pesquisa qualitativa^(18)^.

### Participantes e fontes dos dados

Participaram do estudo 19 profissionais de saúde, sendo 11 enfermeiros e oito médicos, que são atuantes na assistência ao abortamento na Região Metropolitana do estado do Pará, Brasil.

Foi utilizada a técnica de recrutamento denominada *snowball sampling* ^(19)^, conhecida como bola de neve, que consiste em uma técnica não probabilística que não fixa o quantitativo de sujeitos da amostra. Nesse tipo de amostragem, estruturado por meio da primeira semente, direcionado pela pesquisadora, mediante o conhecimento de sua atuação no hospital de referência de abortamento legal em Belém, indicaram-se mais seis profissionais: três médicos e três enfermeiros atuantes na assistência ao abortamento, facilitando o entrosamento do entrevistador com o seu público-alvo^(19)^.

Durante esse processo, cada participante indicado era abordado e convidado por meio do aplicativo *WhatsApp^®^,* e com a resposta positiva, a pesquisadora principal aplicou os seguintes critérios de elegibilidade: enfermeiros e médicos atuantes na assistência à mulher em situação de abortamento. Os critérios de exclusão consideraram profissionais que estavam exercendo como função a gestão. Assim, foi marcada a realização da coleta de dados, ocorrendo remotamente pelo aplicativo *Google Meet^®^.*

No momento da abordagem inicial, fazia-se o convite para cada contato indicado e o estabelecimento de informação sobre o estudo, como tema, objetivos, modalidade de técnicas de coleta de dados, análise, riscos e benefícios. Para aqueles que aceitavam o convite, era marcada a entrevista em dia e horário estabelecidos pelo participante.

Houve apenas esse contato inicial antes da coleta de dados, para fazer o convite e dar explicações acerca do estudo. Não houve nenhuma recusa de participantes; apenas um maior tempo para a realização da coleta de dados, em face do tempo disponibilizado pelo participante, seguindo as recomendações estabelecidas pelo COREQ^(18)^.

### Coleta e organização dos dados

Foi realizada a coleta de dados com a realização da entrevista semiestruturada de forma individual e remota pelo *Google Meet^®^* entre os meses de fevereiro e maio de 2023. Justifica-se a realização da entrevista na forma de coleta de dados, favorecendo a maior disponibilidade de participantes diante dos seus compromissos pessoais e profissionais, especialmente nos da saúde.

A entrevista ocorreu com a presença apenas da pesquisadora principal e do entrevistado, por meio de uma pergunta questionando os participantes se naquele momento estes estavam sozinhos, e, confirmando, era iniciada a entrevista. Cada entrevista teve uma média de 45 minutos de duração. Ocorreu em apenas um único momento, respondendo ao instrumento de entrevista com perguntas abertas e fechadas.

Ressalta-se que foi realizado estudo-piloto com três participantes, que não foram contabilizados no estudo, para verificar a adequação da entrevista aos objetivos da pesquisa e aos depoimentos dos enfermeiros e médicos.

O instrumento continha perguntas de caracterização quanto ao gênero, idade, raça, religião, estado civil, formação acadêmica, ano de formação, se possui especialização, tempo de atuação profissional e no serviço. Também possuía questões norteadoras como: como você percebe a prática do abortamento no âmbito brasileiro? Quais os reais desafios para a saúde da mulher no que se refere ao abortamento? Os depoimentos foram gravados com o recurso de gravação do aplicativo, mediante autorização prévia dos participantes e, posteriormente, transcritas na íntegra para assegurar fidedignidade ao processo de tratamento do material.

Menciona-se que a condução das entrevistas foi realizada apenas pela pesquisadora principal, do gênero feminino, com a seguinte credencial: enfermeira especialista em enfermagem obstétrica. Apenas esta realizou a coleta de dados com o propósito de não haver uma interferência de outros modos de aplicação da entrevista neste estudo. Ressalta-se que a pesquisadora, na época, detinha curso e treinamento das técnicas de pesquisa qualitativa utilizadas no estudo, e estava sendo acompanhada sempre após a coleta de dados com o *feedback* de seu orientador e outros membros da equipe. Eles não fizeram nenhuma interferência no processo de coleta de dados; são doutores de investigação, além de possuírem experiências da aplicação da entrevista em outros estudos. Dessa forma, a equipe de investigação ficou na competência de atuação no tratamento e análise dos dados.

O encerramento da coleta de dados e o estabelecimento do número de participantes do estudo foram feitos por meio do processo de saturação teórica^(20)^. Isto ocorre quando os dados não trazem nenhum elemento novo para balizar ou aprofundar a teorização e o acréscimo de novas informações deixa de ser necessário^(20)^, dando assim um cerne de significado ao problema e atingindo o critério de saturação. Após a realização da 16ª entrevista, foram realizadas mais três coletas e, não obtendo nenhum outro elemento novo representativo, foram considerados saturados os dados do estudo. Trata-se de critério que permite estabelecer a validade de um conjunto de dados, conforme é determinado pelo COREQ^(18)^.

Após a transcrição do material, a pesquisadora fez uma devolutiva do material transcrito em até três dias por meio do *WhatsApp^®^*, garantindo os preceitos de confiabilidade dos dados, como determinam as indicações do COREQ^(18)^, onde não houve nenhum comentário diferente do que foi relatado em relação ao material transcrito.

### Análise dos dados

A análise dos dados foi realizada baseada na técnica de análise de conteúdo^(21)^, visando compreender os discursos das participantes por meio de três etapas cronológicas), a saber: pré-análise das entrevistas, onde ocorreu a organização do material: transcrição de cada entrevista na íntegra; e submissão à leitura flutuante e estabelecimento do contato inicial com os dados do estudo, que buscou na primeira mensagem obtida dos dados, com a escolha de documentos que iriam compor o *corpus* do estudo, de acordo com dados na exaustividade, representatividade, homogeneidade e pertinência^(21)^.

Em seguida, ocorreu a etapa da exploração do material, onde foram construídas as intervenções de codificação. Assim, foi verificado por meio da palavra que se estabeleceu pela técnica de colometria (marcação em distintas cores e legendas o tema central de cada palavra e frase dos depoimentos). Os temas pelo processo podem ser exemplificados a seguir: valor e direito da mulher ao abortamento; valor no âmbito da legislação; valor à vida; valores religiosos, morais e políticos; desconforto em participar do procedimento de abortamento legal; acolhimento e assistência humanizada; e redução da mortalidade materna e dos abortamentos inseguros.

O propósito desta etapa da análise de conteúdo busca a unidade de registro a partir do tema. Dessa forma, estabeleceram-se as unidades de registro com a pertinência da releitura de todo o material e, assim, o agrupamento, a saber: valor e vida; valor religioso e moral; a ética no abortamento; não valor do abortamento; dualidade entre o valor moral e religioso e o valor à vida; valor vital no campo da saúde; mortalidade materna x valor vital^(21)^, que subsidiou a formação de três eixos temáticos e três categorias temáticas, como expressado no Quadro 1.

**Quadro 1:** Estabelecimento das unidades de registro, eixos temáticos e categorias temáticas do estudo

Unidade de registro	Eixo temático	Categoria temática
Valor e vida	Valores da prática profissional para vida das mulheres.	Valores atribuídos por enfermeiros e médicos ao processo de abortamento legal
Valor religioso e moral		
A ética no abortamento		
Não valor do abortamento	O não valor ao aborto.	O não valor ao abortamento na prática profissional
Dualidade entre o valor moral e religioso e o valor à vida		
Valor vital no campo da saúde	A saúde e o valor vital das mulheres.	O valor vital para o abortamento inseguro: uma questão de saúde pública
Mortalidade materna x valor vital		

Na última etapa cronológica do processo analítico, o tratamento dos resultados, inferência e interpretação, classificaram-se os elementos construtivos pelo processo de exclusão mútua, homogeneidade, pertinência, objetividade e fidelidade, e produtividade, visando à categorização^(21)^. Desse modo, os eixos temáticos estabelecidos no processo analítico permitiram a formulação das seguintes categorias temáticas: 1) Valores atribuídos por enfermeiros e médicos ao processo de abortamento legal; 2) O não valor ao abortamento na prática profissional; e 3) O valor vital para o abortamento inseguro: uma questão de saúde pública. As categorias estabelecidas foram discutidas com base no referencial da teoria dos valores de Max Scheler^(14)^ e nas políticas públicas brasileiras referentes à saúde reprodutiva, bem como na literatura especializada sobre o abortamento.

## RESULTADOS

Referente à caracterização dos participantes quanto ao gênero, houve predominância de 15 do gênero feminino e quatro gênero do masculino. Em relação à idade, houve um predomínio de sete participantes maiores de 50 anos, seguidos de cinco entre 31 e 40 anos, quatro entre 21 e 30 anos e três entre 41 e 50 anos.

Em relação à raça autodeclarada, a maioria foi constituída por dez pardos e nove brancos. Em relação à religião, houve predomínio de 12 católicos, seguidos de quatro protestantes, um espírita, um kardecista e um de religião afrodescendente. No estado civil, houve o predomínio de oito casados, seguidos de seis solteiros, quatro em união estável e um divorciado.

Quanto à instituição de formação, 15 participantes eram provenientes de instituições públicas e quatro de particulares. Em relação ao ano de formação, houve um predomínio de nove participantes com formação maior que 2015, seguidos de sete formados entre 2006 e 2015 e quatro formados entre 1995 e 2005.

Todos possuíam especialização *lato sensu* em enfermagem obstétrica e obstetrícia e ginecologia. Sobre o tempo da sua respectiva atuação como enfermeiro ou médico, houve um predomínio de sete participantes entre 5 e 10 anos, seguidos de seis entre 11 e 20 anos, cinco com menos de 5 anos e um maior de 20 anos de atuação na obstetrícia/enfermagem obstétrica.

Em relação ao tempo de atuação no serviço, houve uma predominância de nove com menos de 5 anos, seguidos de cinco com tempo superior a 20 anos, três, entre 11 e 20 anos, e dois, entre 5 e 10 anos de atuação no serviço.

Os dados a seguir ilustram o recorte dos valores atribuídos ao processo analítico do estudo, com a sua consistência metodológica, que está apresentado a seguir:

### Valores atribuídos por enfermeiros e médicos ao processo de abortamento legal

Os valores atribuídos pelos profissionais de saúde mostram que o abortamento legal constitui um direito da mulher, especialmente um valor vital inerente à vida, que deve ser respeitado nas situações amparadas pela legislação brasileira, garantindo a vida das mulheres brasileiras, conforme destacado nos depoimentos a seguir:

*É um direito da mulher em casos de estupro de meninas abaixo de 14 anos, como qualquer mulher. Em casos de risco materno ou anencefalia, a gente ainda está muito atrás disso, porque tem outros casos de malformações fetais incompatíveis com a vida que não estão englobadas.* (E1)

*Significa um direito à vida, um direito que a pessoa tem de não ser apenas uma incubadora, de não ser vista apenas como um objeto que carrega uma vida dentro. São duas vidas, então, para mim, significa olhar para a mulher como ela é, sendo uma vida também e olhar para a situação como duas vidas e não uma vida em um objeto.* (M4)

Os depoimentos dos profissionais de saúde demonstram as suas percepções acerca das discussões sobre o abortamento, seja no âmbito político, seja nas unidades de saúde, seja entre gestores e profissionais. Essas resultam em um modelo de assistência que recebe interferência dos valores morais e éticos, pertencendo a tabus amparados em questões dogmáticas com pautas conservadoras, que são valores contrários à vida das mulheres que necessitam do abortamento previsto na lei, conforme os trechos a seguir:

*As pacientes que buscam o judiciário para ter o respaldo conseguem abortar, mas na assistência, ainda tem alguns entraves. Alguns profissionais não concordam em realizar o procedimento, e postergam o atendimento por questões religiosas, morais e não participam da curetagem no pós-abortamento.* (E4)

*A gente observa hoje, no país, a mortalidade que diz respeito ao atraso no cuidado. A negligência do cuidado se reflete na desigualdade social, econômica e racial, porque mulheres com dinheiro têm assistência com mais qualidade, e quando a gente atua no SUS, percebe o quanto essas desigualdades sociais são marcadas nas mulheres quanto à violência de gênero, discriminação racial, de gênero, homens transgênero abortando, intolerância religiosa e entre outras coisas que vão se refletir em violência de assistência à mulher.* (M2)

### O não valor ao abortamento na prática profissional

É evidente que os profissionais tomam suas decisões influenciados por diversos fatores valorativos, tais como princípios da ética profissional, religião, questões políticas, a compreensão da legislação sobre abortamento e a necessidade de acolher indivíduos em situação de vulnerabilidade. Algumas vezes, essa análise se restringe ao cumprimento do que é estabelecido na jurisprudência. No entanto, muitos profissionais de saúde demonstram desconforto em se posicionar sobre o tema, tratando-o como um assunto polêmico, como evidenciado nos depoimentos a seguir:

*O abortamento é necessário desde que seguida a lei; temos que seguir a norma. Como cristã, acho que todo cristão é contra o abortamento, não é? Olhando pela ótica da religião, mas como profissional, a gente tem que seguir o que é normatizado pela lei. Sou a favor, desde que realmente esteja naqueles critérios estabelecidos em lei.* (E2)

*Eu sou contra o abortamento. É um assunto que está em muita discussão agora, muita coisa querendo ser mudada, mudanças muito radicais. Eu penso que o que está sendo colocado hoje já é alguma coisa que deve ser discutido, e devem ser considerados alguns limites, verificando situações especiais que precisam de mais detalhes para serem permitidas. Penso que ainda seja necessária muita discussão. Mas aborto, liberar todos os tipos, isso eu sou contra.* (M3)

Sobre os profissionais que referiram não participar, apresentam um não valor ao abortamento e a o cuidado com a própria saúde da mulher. Devido a estarem desconfortáveis sobre o assunto, perceberam-se a influência de valores religiosos, o pensamento dualista entre vida e morte, bem como os profissionais que apresentaram ser totalmente contrários em participar do processo de interrupção da gestação por questões legais, frisando maior incômodo em casos resultantes de violência sexual, conforme os depoimentos a seguir:

*O médico não está sendo obrigado a fazer. A gente pode se negar a fazer por questões pessoais, religiosas, e eu me colocaria nessa posição. Eu não concordo, e só naquela situação de risco de vida para mãe, e são raríssimos os motivos pelo quais a gente vai ter que salvar a vida da mãe fazendo um abortamento; só por isso que eu faria. Mas mesmo assim, em último caso, se não tivesse ninguém para fazer no meu lugar, fosse um risco iminente, enfim, não faria.* (M6)

*Eu participaria, dependendo do caso. alguns casos eu não ia me sentir à vontade, uma questão de fé, mas na maioria deles eu participaria, pensando nas mulheres. Por exemplo, essa mulher com um problema de coração e que a gestação é um risco para ela ou uma mulher que está com um bebê malformado incompatível com a vida e que realmente não teria vantagem de se levar a gestação e ela deseja. Já em outros casos, eu preferiria não participar, como em casos nos quais a mãe está bem, o bebê está bem, que poderia ser um bebê de adoção se essa pessoa fosse bem acolhida, encaminhada para a adoção, se fosse possível, eu encaminharia.* (M4)

### O valor vital para o abortamento inseguro: uma questão de saúde pública

Os profissionais de saúde relacionam os abortamentos inseguros a maiores chances de repercussões negativas na saúde das mulheres, como as mortes maternas evitáveis, além do impacto no corpo das mulheres. Os valores vitais são inerentes à saúde das mulheres, visto que uma prática insegura pode repercutir na morte, como mostram os seus desfechos negativos:

*O abortamento inseguro é uma das principais causas de morte da mulher; infelizmente, então, pode trazer um abalo psicológico, um acesso ineficaz aos direitos, às políticas públicas, à rede de atenção à mulher. Então, o abortamento inseguro, para mim, quando chega a acontecer, penso que já vem desencadeado por uma série de fatores: a questão do preconceito, do julgamento social e da falta de educação em saúde da população.* (E7)

*Morbimortalidade, near miss, hemorragia, admissão em UTI, agravamentos sociais, linchamentos, exposição na mídia, viralização. Existe o risco infeccioso e evoluir para sepse, pode ter uma perfuração, risco de se arrepender, esse penso que é o maior, porque ela não tem muita opção de decisão, então ela pode se apoiar em alguma posição ilegal.* (M4)

Os profissionais perpetuam a gravidade e os desfechos negativos que o abortamento inseguro acarreta. Dessa forma, ao se pensar na descriminalização, legalização e ampliação de medidas para o acesso ao abortamento legal no país, especialmente na redução da mortalidade materna, estabelece-se, na sua visão de mundo, um valor vital que, por sua vez, estabelece-se como protetor contra a mortalidade materna por fatores de abortamentos inseguros. Isto ocorre com o devido acesso aos serviços de saúde reprodutiva, conforme os depoimentos a seguir:

*Sou a favor de que seja ampliada e facilitada as formas de aborto legal. A mulher não deve ser obrigada a continuar uma gestação, ela tem direitos de tomar a decisão se quer ou não. A ampliação de políticas de acesso ao abortamento legal é muito importante por conta da saúde da mulher. Se ela não faz legalmente, ela partirá para um inseguro.* (E4)

*Se olharmos para países que conseguiram fazer isso, a gente sabe mais ou menos o que acontecerá, uma diminuição da mortalidade materna provocada por infecção, por hemorragia e provocada por abortamentos inseguros. A gente vê uma maior adesão ao planejamento reprodutivo, e acredito serem as duas principais coisas: redução da mortalidade materna e ampliação dos serviços de planejamento reprodutivo.* (M2)

Desse modo, o acesso ao abortamento legal se torna um valor vital para uma centralidade na vida das mulheres, e sua descriminalização constitui uma importante reflexão prática como marco para a garantia dos direitos sexuais, reprodutivos e humanos na vida das mulheres.

## DISCUSSÃO

Os profissionais de saúde reconhecem que o abortamento legal constitui uma justiça reprodutiva^(12)^ e um valor vital^(14)^ das mulheres, devendo ser assegurado nos dispositivos legais pelo Estado brasileiro. Contudo, a posse do valor para a vida das mulheres e o reconhecimento pelos profissionais de saúde não o efetivam na prática, tornando-se necessário haver uma conscientização para justiça reprodutiva para a concretização do aborto legal. Nesse sentido, depara-se com a inércia de discussões entre profissionais e gestores dos serviços de saúde, bem como a falta de informações às mulheres sobre seus direitos, especialmente na legislação, que garante o abortamento proveniente de violência sexual, risco materno e/ou fetos com anencefalia^(7)^. O que é necessário é a implementação do valor vital do aborto e, se o Estado respeitar essa prática, a vida das mulheres terá um senso de valor garantido^(14)^.

A essência da descrição dos fenômenos se baseia a partir da intuição emocional, na qual o valor vital é compreendido pela emoção com um sentido de valor afetivo. Este é um fator vital que tem valor para a vida das mulheres no âmbito do bem-estar da mulher em situação de abortamento. Assim, o foco de discussão do estudo com base no filósofo discute a relação entre o valor vital^(14)^ e o cuidado na saúde, enfatizando a importância do bem-estar, segurança e vida das mulheres. Segundo a perspectiva axiológica, essa relação é fundamental para a atuação do profissional de saúde^(14)^. Além disso, destaca que qualquer fator que se oponha a essas questões representa uma ameaça à vida das mulheres.

Nesse sentido, no Uruguai, por exemplo, mesmo após a legalização do abortamento, mulheres que procuraram os serviços de aborto legal no setor público relataram sentir-se julgadas pelos profissionais de saúde. Esses resultados evidenciam a importância de sensibilizar e capacitar a equipe de saúde e os serviços especializados com humanização, empatia e escuta atentiva, as quais são estratégias cruciais para reduzir os comportamentos discriminatórios em relação às mulheres que buscam atendimento para o abortamento^(22)^. Apesar da legalização do aborto no Uruguai, ainda ocorrem atos discriminatórios contra as mulheres. Isso perpetua a ideia de que a vida das mulheres não é considerada sob uma dimensão vital ou de valor pelos profissionais de saúde.

Por ser um problema recorrente na mídia, o abortamento se vincula a situações traumáticas experimentadas por mulheres resultantes de intimidações, coações e violência. Isso cria obstáculos para a realização de interrupção da gestação, mesmo quando amparadas legalmente. Esses fatores acentuam as marcas do julgamento moral e preconceituoso, fortemente presentes em discursos de profissionais de saúde. Esses discursos acabam se traduzindo em atitudes inadequadas em sua prática de cuidado com a mulher^(1,2,4,7,8)^.

A Pesquisa Nacional de Aborto revela a recorrência do fenômeno do abortamento entre mulheres de diversas faixas etárias, grupos raciais, níveis educacionais, sociais e crenças religiosas. Esta situação evidencia vulnerabilidades e desigualdades no acesso aos serviços de saúde de qualidade, especialmente para mulheres negras, pardas, indígenas, de baixa renda e escolaridade. Estes são os casos que frequentemente enfrentam óbitos maternos decorrentes de abortos inseguros e clandestinos^(23)^. A desigualdade racial e de classes coloca as mulheres em situação de vulnerabilidade diante da justiça reprodutiva, refletindo na realidade das desigualdades e valor da vida das mulheres ricas, que pagam pelo procedimento, enquanto mulheres pobres e pretas morrem^(24)^.

A dimensão contrária às garantias legais que extrapolam os parâmetros éticos da profissão se tornam verdadeiros obstáculos constantemente vivenciados pelas mulheres brasileiras. Elas buscam o aborto legal, apesar das questões profissionais, da objeção de consciência médica, da negativa por parte da equipe multiprofissional, da desconfiança ante suas alegações, do julgamento e do preconceito. A principal barreira referida pelas mulheres pretas foi o medo de maus-tratos, principalmente nas que declaravam ter provocado o aborto. Além disso, enfrentam obstáculos de acesso aos serviços de saúde, que, em sua maioria, concentram-se em grandes centros urbanos do país, e peregrinação em unidades de saúde. Tais fatores as influenciam a abdicar de seu direito e favorecem a busca de abortamento inseguro e clandestino^(23,25)^.

Estudo^(26)^ afirma que o atendimento ao aborto é um componente essencial do atendimento abrangente à justiça reprodutiva para o cuidado da saúde da mulher, como menciona o Colégio Americano de Obstetras e Ginecologistas. Assim, a relação entre o profissional de saúde e a mulher que busca o serviço para efetivar seu direito deve ser baseada no respeito e na informação. Além disso, eles possuem uma obrigação e um valor ético na prestação do atendimento ao aborto, que tende a incluir o fornecimento de informações, gerenciamento do aborto e atendimento pós-aborto, que resguarda a dignidade e autonomia corporal da mulher.

A discriminação nos serviços de saúde é um problema recorrente para as mulheres que enfrentam situações de abortamento, manifestando-se de maneira direta e indireta. Isso inclui tratamento desumano, julgamento moral e constrangimentos, que frequentemente se manifestam como formas de violência no momento do atendimento a essas mulheres. Essa falta de dignidade no atendimento hospitalar acaba afetando até mesmo mulheres que experienciaram abortos espontâneos, suspeitas injustamente de terem provocado o aborto^(27)^.

Atualmente, observamos um crescente movimento conservador que se opõe aos direitos das mulheres, refletindo uma desvalorização da vida e da segurança das mulheres em situação de abortamento. Segundo a teoria dos valores, o profissional de saúde orienta sua atuação com base em princípios que buscam satisfazer suas necessidades e intuições emocionais, promovendo um sentido de plenitude em sua prática laboral^(14)^. Quando médicos e enfermeiros que atuam na assistência ao abortamento baseiam seus julgamentos em questões moralistas ou religiosas, penalizam a vida das mulheres, tratando-as como se sua vida tivesse menos valor. Isto ocorre mesmo em circunstâncias em que o aborto é garantido, como em casos de risco à vida da mulher, gravidez resultante de estupro ou anencefalia do feto.

Esse contexto gera não apenas discriminação e julgamento por parte da sociedade e dos profissionais de saúde, mas também uma pressão crescente para penalizar essas mulheres pelo simples exercício de seus direitos. O valor moral e ético que se relaciona ao aspecto espiritual do ser humano é fundamental e se apresenta como um fenômeno intrínseco a cada pessoa^(14)^.

Esses valores estão profundamente conectados ao cuidado com as mulheres e ao bem-estar da vida humana, exigindo uma abordagem ética que respeite a dignidade do outro. O valor espiritual, que envolve a vida da mulher, transcende a conduta puramente racional, e é referido pelo filósofo como uma “conduta simpatizante” - um cuidado ético em relação ao próximo. Simpatizar implica uma vivência compartilhada que vai além das emoções efêmeras, manifestando-se como um ato de amor ao outro^(28)^.

Assim, reconhecendo a mulher como a fundação ética que carrega um valor intrínseco, devemos nos empenhar no cuidado genuíno da vida humana. Isso implica perceber a vivência do outro - sua dor e sua luta -, com uma visão integral do ser humano como um indivíduo espiritual^(28)^.

Estudo^(29)^ apontou que os profissionais têm o dever ético, em particular, de cobrar dos Estados a obrigação de respeitar, proteger e cumprir o direito à vida e o direito à saúde, especialmente de tomar medidas para reduzir a mortalidade e a morbilidade materna. Além disso, eles devem garantir que, onde o aborto for considerado legal, que seja seguro e acessível.

A Federação Brasileira das Associações de Ginecologia e Obstetrícia^(30)^ recomendou aos médicos que empreguem todos os esforços para realizar o abortamento permitido por lei em casos de gestações resultantes de violência sexual, priorizando as fases iniciais da gestação e, principalmente, antes das 22 semanas gestacionais. É essencial que os médicos ajam de forma imparcial, acolhedora, sem emitir julgamentos, e forneçam à gestante todas as informações sobre seus direitos. Se não for possível realizar o procedimento no local, a gestante deve ser prontamente encaminhada para outro serviço que tenha as condições necessárias para efetuar o procedimento. Mas, no contexto recente, em 2024, houve uma apresentação de Projeto de Lei nº 1904/2024, com grande repercussão nacional, que prevê que o aborto realizado acima de 22 semanas de gestação, em qualquer situação, passará ser considerado homicídio, inclusive nos casos amparados na legislação brasileira para o aborto^(31)^, em que há um desvelamento para uma prática profissional que tem um contravalor e não valor para as questões inerentes ao abortamento.

Nesse ponto, apesar de considerar o aborto conforme a legislação, a prática profissional revelou o desconforto e não aceitação por parte de alguns médicos e enfermeiros em realizar o abortamento legal por questões relacionadas à dualidade entre vida e morte associada à religião. Assim, foram mais marcantes casos de gestação resultante de violência sexual, com a realização da coação e da ideia de orientar a mulher em prosseguir com a gravidez e encaminhar posteriormente o bebê para adoção. Tal movimento se distancia das diretrizes de acolher e orientar com imparcialidade. Assim, cabe ao profissional de saúde adotar uma atitude terapêutica, sendo terminantemente proibido a imposição de seus valores durante o acolhimento à mulher e na prestação de orientação sobre o abortamento. Também não se deve enfatizar indevidamente supostos riscos que não correspondam à realidade, na tentativa de coagir a mulher a repensar sua decisão^(4)^.

Quando há esse desvelar fenomenológico pelo ato de contravaloração e não valor por parte dos profissionais, que são contrários a qualquer questão oriunda do abortamento ou não detêm uma posição formada sobre o tema, repercute em um contravalor e não valor à vida e à pessoa humana, que neste caso se configura na mulher^(14)^.

O profissional de saúde que não compactua com a escolha da mulher no abortamento viola as obrigações éticas centrais da profissão, como autonomia, beneficência, não maleficência e justiça. A obrigação de respeitar e promover a autonomia da mulher, o eixo da ética médica moderna, é uma justificativa central para o amplo dever ético dos clínicos de fornecer informações de saúde^(26)^. Dessa forma, médicos e enfermeiros têm a sua obrigação de garantir a informação do abortamento e respeitar a escolha da mulher, tendo a sua vida como o valor primordial a ser assegurado.

Com base nas estatísticas da OMS^(4)^, são realizados, anualmente, cerca de 25 milhões de abortos inseguros em todo o mundo. Em áreas onde a prática é legalizada, a taxa de interrupções inseguras gira em torno de 10%. No entanto, em regiões onde o aborto é criminalizado, esse número salta para 25%. É alarmante estimar que aproximadamente 39 mil mulheres são hospitalizadas ou falecem a cada ano devido a complicações decorrentes de abortos inseguros. É crucial ressaltar que a grande maioria dessas tragédias poderia ter sido evitada caso fossem realizadas por profissionais qualificados e de acordo com métodos recomendados. Os abortos inseguros e clandestinos não apenas representam uma séria preocupação para a saúde pública, mas também emergem como uma das principais causas de mortalidade materna em todo o mundo, conforme destacado pela OMS^(32)^.

A saúde pública se configura como um desvelar para a saúde das mulheres, e neste sentido, tem um valor vital, cuja essência é a vida para a pessoa humana. Assim, a criminalização traz consigo riscos substanciais para a saúde mental das mulheres. Estes riscos advêm das gestações não planejadas, que podem levar a interrupções por meio de abortos inseguros, contribuindo para a sua morte e não valorização de sua pessoa com essência na vida. Além disso, existe uma maior propensão para desenvolver depressão pós-parto e adotar comportamentos menos saudáveis durante a gestação. Estes comportamentos podem acarretar complicações que afetam, inclusive, os futuros filhos^(33)^.

Exemplo de equidade e justiça reprodutiva, no Canadá, o aborto é reconhecido e regulamentado como assistência médica. A legalidade dos serviços de aborto não está sujeita à mudança do clima político ou às mudanças de atitudes sociais. Neste sentido, politizar o tema traz uma forma errônea sobre o aspecto de equidade à saúde e, neste país, ecoa um profundo respeito pela autonomia pessoal e integridade médica, garantindo que o aborto, como um serviço de saúde, permaneça acessível e adaptável^(34)^. Dessa forma, o Brasil tem muito a aprender e tem como exemplo essa iniciativa para as mulheres brasileiras e a prática dos profissionais de saúde.

Sobre a descriminalização do abortamento ser um caminho que possibilita uma prática de cuidado integralizada e segura às mulheres, os profissionais demonstram concordâncias e perspectivas positivas diante da redução da mortalidade materna e ampliação do acesso a serviços de saúde reprodutiva. Países que possuem leis acerca do abortamento menos restritivas apresentaram redução na mortalidade materna, ampliação da contracepção, oportunidade de aconselhamento e inserção crescente de métodos de longa duração, como o DIU, após o procedimento de interrupção da gestação, sendo bem aceito pelas mulheres. Tais resultados demonstram os impactos favoráveis com a legalização do abortamento, garantindo maior acessibilidade no sistema de saúde^(24,35,36)^.

As diretrizes recentes apresentadas pela OMS^(4)^ no guia de aborto seguro destacam o atraso e a lentidão com que o Brasil está progredindo em direção à descriminalização do aborto e à expansão do acesso aos serviços de saúde. Enquanto isso, uma onda de legalização do aborto está ganhando força em países vizinhos.

Dessa forma, a acessibilidade ao aborto legal e sua descriminalização representam um marco crucial na garantia da justiça reprodutiva com equidade social para as mulheres. É de extrema urgência a luta por progressos nessas questões no Brasil, envolvendo não apenas a sociedade em geral, mas também os serviços de saúde, seus administradores e os profissionais envolvidos. Oferecer informações, disponibilizar serviços especializados e promover o planejamento reprodutivo são estratégias essenciais para a redução dos casos de aborto inseguro no país e da mortalidade materna. Torna-se imperativo que haja educação sexual como meio de tomada de decisão, adoção de métodos contraceptivos para evitar o aborto e disponibilidade do aborto seguro para prevenir óbitos.

Desse modo, há necessidade de garantir a saúde da mulher como uma questão de valor. Quando o profissional de saúde se expressa em favor dos direitos sexuais e reprodutivos, isto permite, pelo ato do seu perceber emocional, garantir um valor vital, um valor moral e ético para o cuidado da pessoa humana e, neste caso, a mulher livre de qualquer malefício proveniente de um ato inseguro.

### Limitações do estudo

O estudo teve como limitação a utilização de outras técnicas de coleta, como observação, por conta de a entrevista ocorrer de forma *online*.

### Contribuições para as áreas da enfermagem, saúde ou políticas públicas

O estudo contribui para o conhecimento acerca dos valores atribuídos por médicos e enfermeiros obstétricos ao abortamento legal, mediante fatos que hoje perpassam pelos valores vitais (para a vida), éticos-profissionais e morais das mulheres. Esses valores são fundantes na sua atuação profissional, no sentido de visão de mundo, que repercute diretamente na sua prática.

## CONSIDERAÇÕES FINAIS

O presente estudo teve em vista compreender os valores atribuídos por enfermeiros e médicos que atuam na assistência obstétrica em relação ao abortamento. Os resultados revelaram um discurso que abrange aspectos vitais, morais e éticos no contexto da saúde das mulheres. Para os profissionais de saúde, a saúde e o bem-estar físico das pacientes são valores fundamentais, os quais exercem influência direta na sua prática assistencial.

Por outro lado, também existem contravalores e ausência de valores na formação filosófica sobre o tema. O abortamento, frequentemente, é visto como um indicativo de falta de uma posição afirmativa, impactando diretamente a vida das mulheres, especialmente em relação à mortalidade materna.

Em um Estado laico como o Brasil, a discussão sobre o abortamento - considerado um grave problema de saúde pública quando realizado de forma insegura - não deveria ser guiada por questões religiosas e morais, visto que isso redireciona o debate para caminhos inadequados. É imperativo garantir justiça reprodutiva e equidade social, promovendo a inclusão desse tema nas políticas públicas. O abortamento deve ser encarado como uma estratégia para valorizar a vida das mulheres e combater a mortalidade materna.

Essa dinâmica evidencia os dilemas morais e éticos enfrentados pelos profissionais de saúde em relação à interrupção legal da gestação. Enquanto alguns reconhecem a importância de oferecer assistência pautada no acolhimento e na humanização, outros encontram justificativas para se abster desse papel. Muitas vezes, a prática desses profissionais não prioriza o direito da mulher, mas, sim, um contexto de julgamento, coação e discriminação em relação às suas decisões sobre o abortamento legal.

Dessa forma, a descriminalização do abortamento no Brasil torna-se um tema crucial a ser pesquisado, requerendo o apoio de profissionais de saúde, gestores, políticos e da sociedade na totalidade, uma vez que o abortamento inseguro no país contribui para elevados índices de mortalidade materna. Portanto, estudos que investiguem tanto o abortamento legal, sua descriminalização como sua legalização são essenciais para um entendimento mais amplo sobre essa questão.
